# Current progress and future perspectives of neoadjuvant anti-PD-1/PD-L1 therapy for colorectal cancer

**DOI:** 10.3389/fimmu.2022.1001444

**Published:** 2022-09-09

**Authors:** Zhengyang Yang, Guocong Wu, Xiao Zhang, Jiale Gao, Cong Meng, Yishan Liu, Qi Wei, Liting Sun, Pengyu Wei, Zhigang Bai, Hongwei Yao, Zhongtao Zhang

**Affiliations:** Department of General Surgery, Beijing Friendship Hospital, Capital Medical University and National Clinical Research Center for Digestive Diseases, Beijing, China

**Keywords:** colorectal cancer, PD-1/PD-L1 inhibitors, neoadjuvant, microsatellite instability, mismatch repair

## Abstract

Immunotherapies, especially the programmed cell death 1/programmed cell death ligand 1 (PD-1/PD-L1) inhibitors, have revolutionized the therapeutic strategies of various cancers. As for colorectal cancer (CRC), the current clinical application of PD-1/PD-L1 inhibitors are mainly used according to the mutation pattern, which is categorized into deficient mismatch repair (dMMR)/high levels of microsatellite instability (MSI-H) and proficient mismatch repair (pMMR), or non-high levels of microsatellite instability (non-MSI-H). PD-1/PD-L1 inhibitors have been proven to have favorable outcomes against dMMR/MSI-H CRC because of more T-cell infiltration into tumor tissues. Nevertheless, the effectiveness of PD-1/PD-L1 inhibitors in pMMR/non-MSI-H CRC is still uncertain. Because of the quite-lower proportion of dMMR/MSI-H in CRC, PD-1/PD-L1 inhibitors have been reported to combine with other antitumor treatments including chemotherapy, radiotherapy, and targeted therapy for better therapeutic effect in recent clinical trials. Neoadjuvant therapy, mainly including chemotherapy and radiotherapy, not only can reduce clinical stage but also benefit from local control, which can improve clinical symptoms and the quality of life. Adding immunotherapy into neoadjuvant therapy may change the treatment strategy of primary resectable or some metastatic CRC. In this review, we focus on the development of neoadjuvant anti-PD-1/PD-L1 therapy and discuss the future perspectives in CRC.

## Introduction

Colorectal cancer (CRC) is one of the most common malignant tumors all over the world, with new cases accounting for 10.0% of all cancers each year (1). At present, the treatment strategies mainly include surgical resection, chemotherapy, radiotherapy, and molecular targeted therapy (2, 3). Although a variety of therapeutic strategies have made significant progress in CRC treatment recently (4–6), the number of CRC-related deaths still reaches 915,880 each year, accounting for 9.4% of all tumor-related deaths, ranking second in all tumors worldwide (7). Consequently, the benefits of current treatment have encountered a bottleneck and novel strategies are urgent for better therapeutic effects in CRC patients.

Recently, immunotherapy has received rapid development and more attention in clinical application because of its good antitumor effect, which further provides motivation for CRC (8, 9). Compared with traditional treatments, immunotherapy could kill cancer cells by activating the antitumor immunity and is specifically targeted against cancer antigens to prevent normal cells from being attacked (10–12). Among them, programmed cell death protein 1 (PDCD1, PD-1) is the most important receptor for activating T-cell expression and mediating immunosuppression, while the programmed cell death ligand 1 (CD274, PD-L1) is involved in programmed death 1, resulting in T-cell apoptosis or anergy (13, 14). Therefore, PD-1/PD-L1 inhibitors could stop T-cell apoptosis and dysfunction, which further enhances the activation of T cells (15). Since nivolumab was firstly used in humans in 2006, PD-1/PD-L1 inhibitors were applied in many clinical trials to treat various refractory cancers, including melanoma, gastric cancer, and lung cancer (16–18). CRC is categorized into deficiency mismatch repair/high levels of microsatellite instability (dMMR/MSI-H) and proficient mismatch repair/non-high levels of microsatellite instability (pMMR/non-MSI-H) according to the mutation pattern (19, 20). Many clinical trials have proven that immune checkpoint inhibitors (ICIs) exhibited effective and stable therapeutic effects on dMMR/MSI-H CRC patients; therefore, several drugs like nivolumab and pembrolizumab are approved by the US Food and Drug Administration to treat this kind of patients (21–23).

Neoadjuvant therapy is the use of radiotherapy, chemotherapy, and a combination of various treatment methods before surgery, which can reduce the staging of tumors, thereby reducing local recurrence and acquiring better prognosis (24–26). At present, neoadjuvant therapy has been proven to be effective in the treatment of some CRC patients, especially locally advanced rectal cancer (LARC) and colorectal liver metastases (CRLMs) (27, 28). Therefore, the overall survival (OS) rate of neoadjuvant therapy is proven to be not remarkably higher than postoperative therapy (29, 30). Neoadjuvant radiotherapy could enlarge the anti-PD-1/PD-L1 treatment effect by promoting different links in the immune response such as the activation and recruitment of T cells, promotion of dendritic cell maturation, antigen exposure, and upregulation of major histocompatibility complex molecules (31, 32). Additionally, neoadjuvant chemotherapy could induce PD-1/PD-L1 expression and further profit the effect of ICI treatment (33, 34). Consequently, adding anti-PD-1/PD-L1 therapy into neoadjuvant therapy might change the treatment strategy of primary resectable or some metastatic CRC and further acquire better prognosis and survival results. Hence, this review aimed to focus on the development of neoadjuvant anti-PD-1/PD-L1 therapy and discuss the current opportunities and challenges, highlighting considerations for the upfront treatment in resectable and part of metastatic CRC.

## Mechanisms of programmed cell death 1/programmed cell death ligand 1 inhibitors in deficient mismatch repair/high levels of microsatellite instability colorectal cancer

### Mechanisms of anti-PD-1/PD-L1 therapy

**Figure 1:**
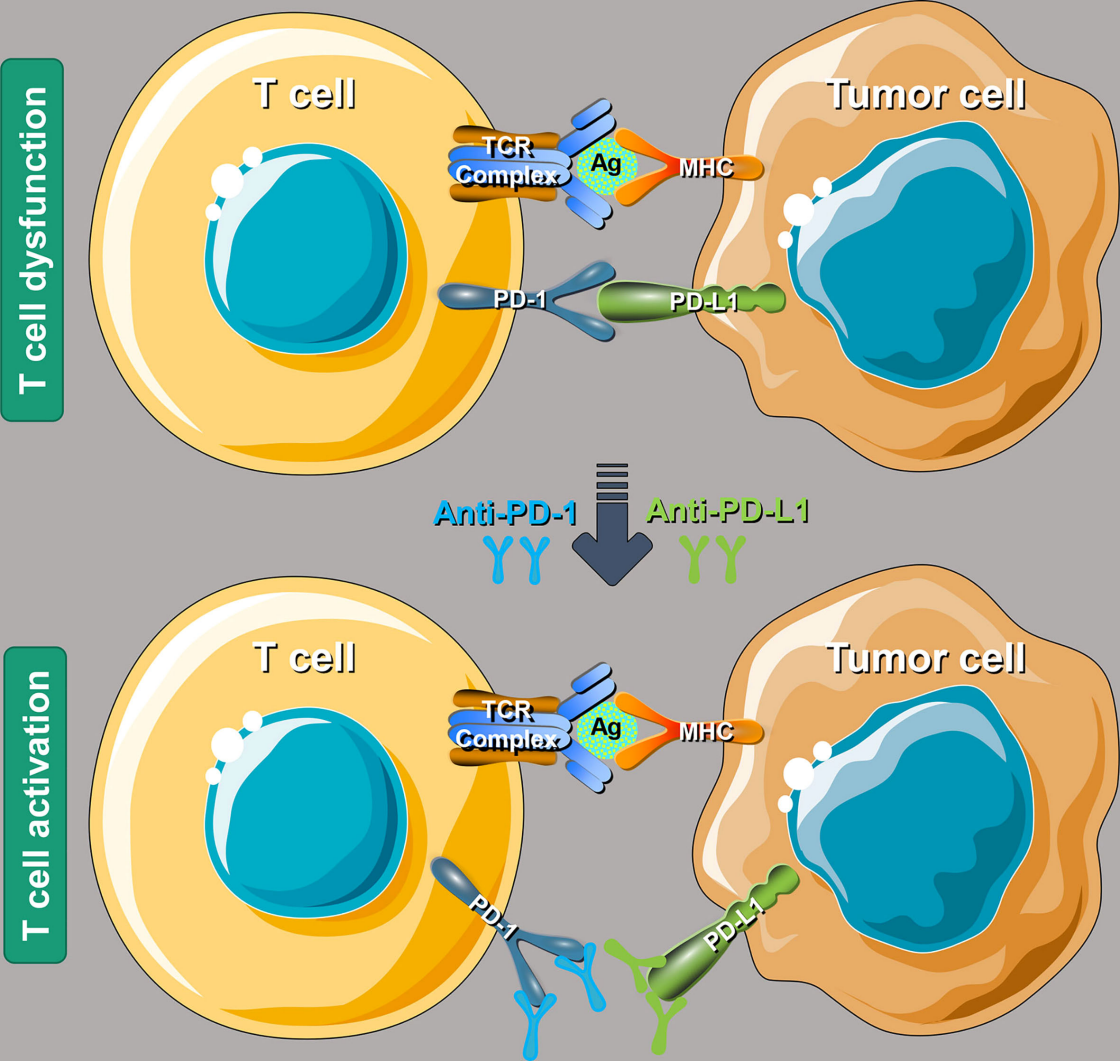
Schematic mechanism of programmed cell death 1/programmed cell death ligand 1 (PD-1/PD-L1) inhibitors to restore T-cell functions. T-cell receptor, antigen, and major histocompatibility complex (MHC). Reproduced with permission (40).

The antitumor immune process mainly includes immune elimination, immune balance, and immune escape. PD-1 and PD-L1 are a pair of important immune checkpoint (ICs) that work as the brake on the immune system and play a crucial role in the tumor immune escaping process (35). After the binding of PD-1 and PD-L1, tumor cells take advantage of the recognition of the T-cell receptor, further suppressing immunity and evading immune surveillance (36). In 2002, the evidence that the PD-1 pathway mediating tumor immunity was first reported in that the overexpression of PD-L1 will weaken the cytolytic activity of T cells and then significantly promote the occurrence and invasion of tumors (37). Interestingly, such effect could be reversed by the application of monoclonal antibodies against PD-L1 (38). PD-L1 is highly expressed on the surface of many tumor cells, which can also induce immune cells [especially T helper lymphocytes, (Th)] to secrete immunosuppressive factors and further inhibit the killing effect of the antitumor immunity (39). As shown in **Figure 1**, anti-PD-1/PD-L1 therapy can bind to PD-1 and PD-L1 correspondingly, further preventing the combination of PD-1 on the surface of T cells and PD-L1 on the surface of tumor cells (40). Such function could reverse the inhibitory effect of the immune system by tumor cells and restore the antitumor immunity.

### Biological features of deficient mismatch repair/high levels of microsatellite instability colorectal cancer

A human body could maintain genomic stability by evolving sophisticated mechanisms. Mismatch repair (MMR) is an evolutionarily conserved system consisting of five key proteins: mutL homolog 1 (MLH1); postmeiotic segregation 2 (PMS2); and mutS homologs 2, 3, and 6 (MSH2, 3, and 6), which could be used to identify and repair base misinsertion, deletion, and misfusion during the progress of DNA replication, DNA recombination, and some forms of DNA damage (41, 42). In addition, MMR can also play a key role in response to DNA-damaging agents by apoptosis induction or regulating the cell cycle (43, 44). Significantly, the change of the MMR status may lead to different microsatellite lengths called microsatellite instability (MSI), which can be accurately detected by PCR or second-generation sequencing technology (45).

During the carcinogenesis of normal colorectal epithelial cells, one of the important driving factors is genomic instability, which results in unrestricted proliferation and the avoidance of immune clearance in cancer cells (46). The state of dMMR/MSI-H in CRC was firstly reported in the Lynch syndrome, which is a kind of inherited cancer syndrome and mainly resulted by the mutations of the MMR gene (most commonly MLH1 and MSH2) (47). Compared with pMMR, dMMR CRC has higher tumor mutational burden (TMB), while the mutation rate increases approximately 100–1,000 times (48). The accounts of the dMMR/MSI-H of all CRC cases are approximately 15% while approximately 85% of patients are proficient in MMR (49). Interestingly, approximately 20% of stage II and 11% of stage III tumors are dMMR/MSI-H; however, the percentage is only 5% in stage IV (50).

The prognosis of stage II or III dMMR/MSI-H CRC patients is significantly better than that of pMMR/non-MSI-H, whereas, stage IV patients were reported with a poor prognosis (51). CRC with dMMR have many noteworthy characteristics like a lymphocytic infiltrate, tendency to arise in the proximal colon, lower transfer rate, and signet ring or mucinous appearance (52). According to previous literature reports, stage II dMMR/MSI-H CRC patients cannot benefit from adjuvant chemotherapy based on traditional cytotoxic drugs like 5-FU (53, 54). This phenomenon might be mainly because dMMR/MSI-H CRC cannot achieve the recognition of 5-FU-modified DNA, which is an important step that triggers the cytotoxic progress (55). However, the efficacy of oxaliplatin adjuvant therapy does not appear to be affected by the MMR or MSI status (56, 57). It has been mainly reported that dMMR/MSI-H CRC has a good response to ICI treatments, especially anti-PD-1/PD-L1 therapy. It is reported that dMMR/MSI-H CRC had remarkably higher levels of cytotoxic T lymphocytes (CTLs), Th1, Th2, follicular helper T cells, and T-cell markers (58). Additionally, higher TMB, tumor neoantigen burden (TNB), and more lymphocyte infiltration and PD-L1 expression in tumor tissues have also been reported (59, 60). The sufficient evidence above prompts that PD-1/PD-L1 inhibitors could enhance antitumor immunity and result in an excellent therapeutic effect when treating these individuals.

### Exploration of programmed cell death 1/programmed cell death ligand 1inhibitors in metastatic deficient mismatch repair/high levels of microsatellite instability colorectal cancer

The KEYNOTE-016 study reported in 2016 that the overall response rate (ORR) was 0% and the disease control rate (DCR) was 16% in pMMR/non-MSI-H CRC patients who received pembrolizumab, compared with 50% and 89% for dMMR/MSI-H, respectively (61). The subsequent phase 2 study, KEYNOTE-164, reported the median PFS of 4.1 months, 24-month OS rate of 63%, ORR of 33%, and DCR of 57% in dMMR/MSI-H metastatic CRC (mCRC) patients (62). CheckMate-142 was a phase 2 clinical trial that evaluated the curative effect of another PD-1 inhibitor, nivolumab, in dMMR/MSI-H mCRC patients. At a median follow-up of 12 months, 31% (23 of 74) of patients reached the ORR, while the OS and progression-free survival (PFS) were 73% and 50%, correspondingly (63). In consideration of the above outcomes, nivolumab and pembrolizumab received the accelerated approval of the FDA as the second-line treatment for patients with dMMR/MSI-H mCRC in 2017 (64).

As an important milestone in the development of CRC immunotherapy, KEYNOTE-177 compared the efficacy of pembrolizumab compared to standard chemotherapy in the first-line treatment of dMMR/MSI-H mCRC. At the final analysis in 2021, median OS (the median follow-up of 44.5 months) was not reached in the pembrolizumab group while it was 36.7 months in the chemotherapy group. In addition, the median PFS was 16.5 months in the pembrolizumab group while it was 8.2 months in the chemotherapy group (65). Due to the gratifying results, pembrolizumab or nivolumab, alone or in combination with ipilimumab, was recommended as a first-line treatment option for patients with dMMR/MSI-H mCRC, whether it is eligible for intensive therapy in National Comprehensive Cancer Network (NCCN) Guidelines Version 2.2021 (66).

## Programmed cell death 1/programmed cell death ligand 1 inhibitors for neoadjuvant treatment in colorectal cancer

Neoadjuvant therapy for CRC mostly focuses on locally advanced rectal cancer and some resectable metastatic CRC. Traditional neoadjuvant therapies include chemotherapy, radiotherapy, targeted therapy, and combination therapy. At present, the neoadjuvant treatment for rectal cancer is based on radiotherapy and combined with chemotherapy drugs, while for colon cancer, it is mostly based on drugs, including chemotherapy drugs and targeted drugs.

### Neoadjuvant anti- programmed cell death 1/programmed cell death ligand 1 therapy in deficient mismatch repair/high levels of microsatellite instability colorectal cancer

NCCN Guidelines Version 2.2021 changed the previous recommendation on detecting the MMR/MSI status. The guidelines recommend universal MMR or MSI testing for all patients with a personal history of colon or rectal cancer. In addition to its role as a predictive marker for immunotherapy use in the advanced CRC setting, the MSI/MMR status can also help to identify individuals with the Lynch syndrome and to inform adjuvant therapy decisions for patients with stage II CRC (66). Previous recommendations limited such testing to patients with suspected metastases. Consequently, new guidelines mean that anti-PD-1/PD-L1 therapy not only can be applicable to stage IV dMMR/MSI-H mCRC patients but also be used as part of neoadjuvant therapy. As mentioned previously, dMMR/MSI-H patients are resistant to some conventional chemotherapy. A retrospective study in 2020 involving 5,086 LARC patients between 2010 and 2015 in the National Cancer Database suggested that the postoperative pathologic complete response (pCR) rate of dMMR/MSI-H after neoadjuvant chemoradiotherapy was significantly lower than that of the pMMR/non-MSI-H group (5.9% vs. 8.9%) (67).

The encouraging results of ICIs in the treatment of dMMR/MSI-H mCRC have greatly promoted the exploration of them in neoadjuvant therapy. The NICHE clinical trial from the Netherlands is the pioneer with the inclusion criteria of stage I, II, or III resectable colon adenocarcinoma (68). Patients with non-metastatic resectable dMMR or pMMR CRC received a single dose of ipilimumab and two doses of nivolumab, followed by surgery within 6 weeks. In addition, patients with pMMR tumors were randomized to receive or not receive celecoxib. Pathological responses (PR, at least 50% tumor regression) were observed in all 20 dMMR patients, including 19 major pathological responses (MPRs, ≤10% residual viable tumor) and 12 pCR. However, 4/15 of pMMR patients reached PR, with three MPRs and no pCR. A phase 2 study from China involved clinical stage T3/T4 or any T with lymph node positivity (N+) dMMR/MSI-H CRC and treated using toripalimab on day 1, with or without celecoxib 200 mg orally twice daily from day 1 to 14 of each 14-day cycle, for six cycles before surgical resection (69). The pCR rate in the toripalimab monotherapy group was 65% (11/17), while in the toripalimab-plus-celecoxib group, it even reached 89% (17/19). A very recent study reported a combination of neoadjuvant chemoradiotherapy and immunotherapy treating dMMR/MSI-H stage II or III rectal cancer (70). Patients received neoadjuvant dostarlimab every 3 weeks for 6 months (nine cycles) and then followed by standard radiation therapy with a concurrent administration of capecitabine at standard doses, and finally followed by total mesorectal excision (TME). All 12 patients who reached a clinical complete response (cCR) have undergone at least 6 months of follow-up, with no evidence of tumor according to magnetic resonance imaging (MRI), ^18^F-fluorodeoxyglucose (FDG) PET, endoscopic evaluation, digital rectal examination, or biopsy. In summary, dMMR/MSI-H CRC receiving neoadjuvant anti-PD-1/PD-L1 therapy could obtain a higher pCR or cCR rate, which might guide clinicians to choose neoadjuvant treatment in the future.

### Neoadjuvant anti-programmed cell death 1/programmed cell death ligand 1 therapy in proficient mismatch repair/non-high levels of microsatellite instability colorectal cancer

**Figure 2:**
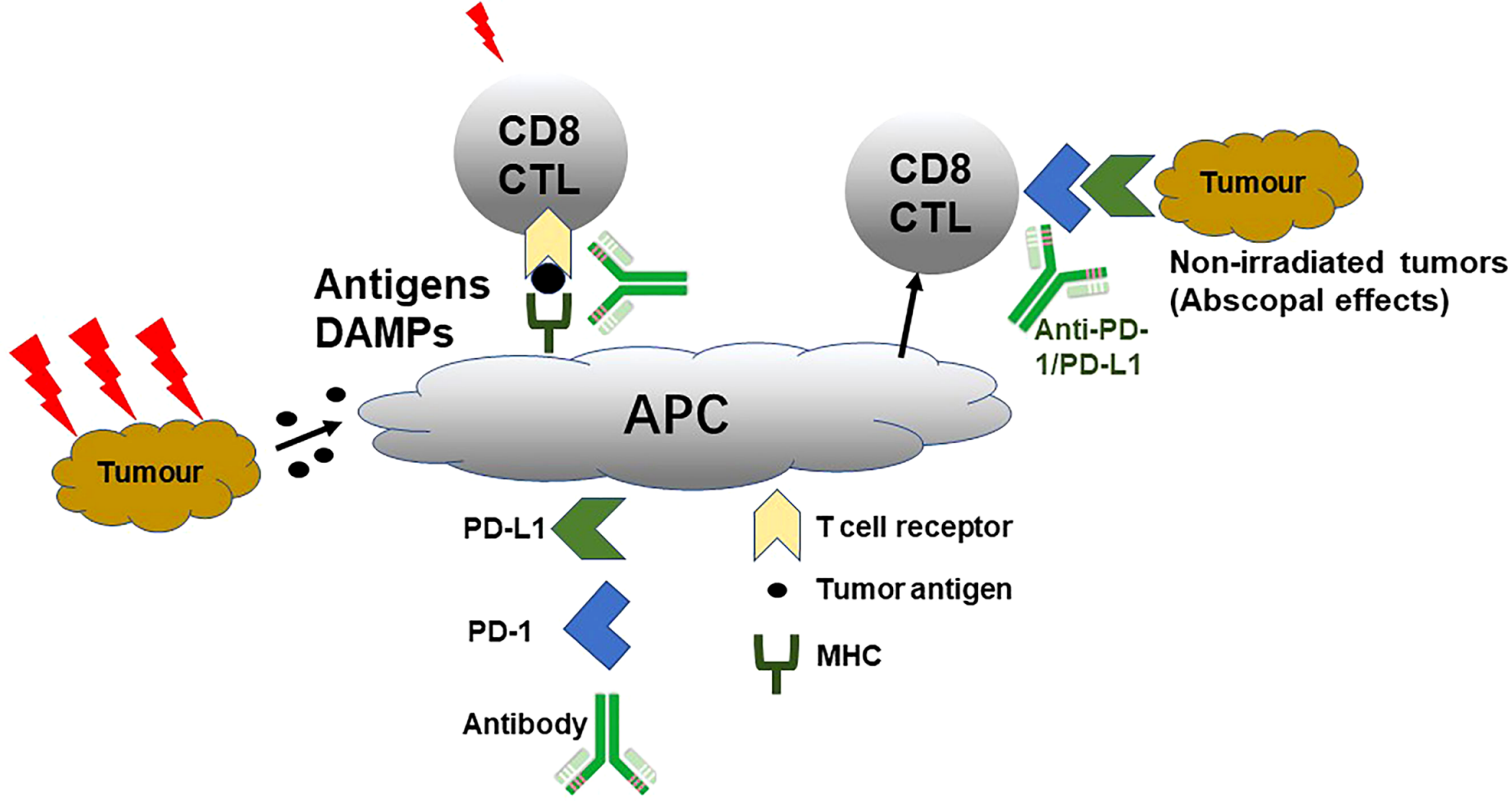
Schematic mechanism of radiotherapy enlarging anti-PD-1/PD-L1 curative effect. Damage-associated molecular patterns, cytotoxic T lymphocytes, antigen-presenting cell, and MHC. Reproduced with permission (72).

Differently, pMMR/non-MSI-H CRC could not respond well to immunotherapy. For this problem, many studies concentrated on the strategy of combined with chemotherapy or radiation therapy to improve the curative effect. Many traditional chemotherapeutic agents like oxaliplatin, 5-FU, and gemcitabine can modulate tumor-infiltrating lymphocytes (TILs) as immunogenic cell death inducers to reactivate antitumor immunity in the tumor-immunosuppressive microenvironment (71). Hence, the combination of chemotherapy and immunotherapy can promote the immune response, enhance the therapeutic effect of ICIs, and further achieve the effect of improving the clinical prognosis of patients. It has been widely demonstrated that radiotherapy combined with immunotherapy could achieve an effect of 1 + 1 > 2 in clinic. As shown in **Figure 2**, radiotherapy can effectively activate the antitumor effect by inducing tumour antigen release, enhancing tumour cell immunogenicity, activating immune cells, and secreting immune factors and promote tumor-related antigen presentation (72). Additionally, radiotherapy not only can upregulate the expression of PD-1 on T cells and PD-L1 on tumor cells for suppressing immunotherapy resistance but also kill tumor cells and induce the release of inflammatory cytokines, damage-associated molecular patterns, and tumor-associated antigens, achieving the synergistic antitumor effect (73, 74).

**Figure 3:**
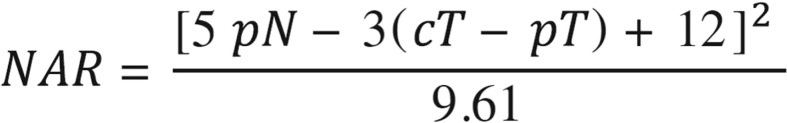
Calculation formula of the neoadjuvant rectal (NAR) score. NAR, pathologic nodal stage, clinical tumor stage, and pathologic tumor stage.

The VOLTAGE-A study from Japan reported the short-term results of T_3–4_N_0-2_M_0_ LARC patients regardless of the MMR/MSI status receiving preoperative immunotherapy combined with chemoradiotherapy followed by radical surgery (75). The detailed neoadjuvant schedule was five cycles of nivolumab after 50.4 Gy with capecitabine. In this study, 11/37 (30%) of MSS patients reaching pCR and 14/37 (38%) reaching MPRs according to the American Joint Committee on Cancer guidelines for the evaluation of the tumor regression grade were observed. As of December 2020, with a median follow-up of 32.9 months, two cases of local recurrence and four cases of distant metastasis were observed in the MSS group. In addition, this study reported a combination of biomarkers (PD-L1 expression in ≥1% of tumor cells, CD8^+^ T-cell/effector regulatory T-cell ratios ≥2.5) to predict the efficacy of neoadjuvant chemoradiotherapy combined with anti-PD-1/PD-L1 therapy in MSS LARC patients, which has good application potential in subsequent studies. A phase 2 single-arm trial from China involved T_3-4_N_0_M_0_ or T_1-4_N_0-2_M_0_ rectal adenocarcinoma (an inferior margin of 10 cm from the anal verge) patients to monitor the outcomes (76). The eligible patients received short-course radiotherapy (5 × 5 Gy over 5 days), followed 1 week later by two subsequent 21-day cycles of CAPOX (oxaliplatin day 1 and capecitabine day 1–14) plus camrelizumab (day 1), followed by radical surgery according to TME principles. The pCR (ypT0N0) rate in pMMR patients reached an amazing 46.2% (12/26). This scheme not only can shorten the preoperative treatment time but also acquired the satisfactory anal preservation rate of 88.9%. An American trial reported in 2021 assessed whether the addition of pembrolizumab to neoadjuvant chemoradiotherapy can lead to an improvement in the neoadjuvant rectal (NAR) score instead of pCR compared with 5-fluorouracil, leucovorin, and oxaliplatin (FOLFOX) (77). As shown in **Figure 3**, the NAR score is calculated according to the following formula as a predictive indicator of survival after preoperative chemoradiotherapy for rectal cancer (78). Patients with stage II/III LARC with distal location (cT_3-4_ , ≤ 5 cm from anal verge, N_0-2_), with bulky disease (any cT_4_ or tumor within 3 mm of mesorectal fascia), at high risk for metastatic disease (cN_2_), and/or who were not candidates for sphincter-sparing surgery (SSS) were enrolled. A total of 185 patients were randomized (1:1) to neoadjuvant FOLFOX for 4 months and then underwent chemoradiotherapy (capecitabine, 50.4 Gy) with (n = 90) or without (n = 95) pembrolizumab (six doses every 3 weeks) before surgery. Unfortunately, this study yielded negative results with the mean NAR score being 11.53 vs. 14.08, cCR rate of 13.9% vs. 13.6%, and pCR rate of 31.9% vs. 29.4% in the pembrolizumab arm and control arm, correspondingly.

### Exploration of neoadjuvant anti-programmed cell death 1/programmed cell death ligand 1 therapy in our center

Our center also initiated a prospective, multicenter, phase 2 clinical trial to explore safety and efficacy of long-course neoadjuvant chemoradiotherapy plus tislelizumab followed by TME for LARC (78). As of 30 June 2022, a total number of patients (n = 43) were enrolled, while 30 (29 pMMR/non-MSI-H and 1 dMMR/MSI-H) patients had undergone TME surgery, with the R0 resection rate of 100% and sphincter-saving resection rate of 90.0% (27/30). The objective response rate reached 100% (30/30) with the pCR rate of 43.3% (13/30) and MPR rate of 40.0% (12/30). At present, this study continues to enroll patients and is estimated to enroll 50 patients. We also expect exciting results at the final primary endpoints (pCR rate) and secondary endpoints (NAR score, ORR, R0 resection rate, anal preservation rate). According to several existing research data, the pCR rate of pMMR/non-MSI-H patients can reach up to 46.2% after combined immunotherapy, which seems to be significantly improved after combined radiotherapy. This strategy may improve the quality of life for LARC patients, especially those with ultralow rectal cancer (≤5 cm from the lower edge of the tumor to the anus), to achieve organ preservation and the use of watch and wait for the future.

## Endpoint evaluation of neoadjuvant immunotherapy

As more and more patients reached pCR in neoadjuvant immunotherapy, Watch and Wait strategy is a strategy which is more and more likely to achieve the purpose of anus reservation and reduce surgical trauma without affecting the survival rate. evaluated at or near cCR can be considered for Watch and Waiting under the premise of close follow-up, while for patients with a clear tumor residue, radical surgery is recommended as soon as possible. Rectal MRI is currently an important staging method recommended by international guidelines for the diagnosis of primary rectal cancer. MRI can accurately display the anatomy of rectum and adjacent organs, further providing relatively accurate information on the tumor stage. However, since the measurement of the tumor site after neoadjuvant therapy is often interfered by necrosis and other factors, traditional MRI methods cannot accurately monitor the tumor response (79).

Due to immune cell infiltration and other reasons, one of the characteristics of immune neoadjuvant therapy is that the imaging and pathological evaluation results may differ greatly. Such a phenomenon is called pseudoprogression (PSPD), which is manifested in that many patients do not observe tumor remission on imaging but maintaining stability or even some enlargement, but a pathological examination may find a tumor regression in these patients (80). Thus, how to recognize and identify the different between PSPD and true progression is significant. An interesting clinical study that included 123 patients with dMMR/MSI-H mCRC treated with ICIs was reported to evaluated the PSPD frequency with the median follow-up of 22.3 months (81). A total of 29% (36/123) of patients experienced radiological progressive disease (PD) according to Response Evaluation Criteria in Solid Tumours, version 1.1 (RECIST 1.1), of which 61.1% (22/36) occurred in the first 3 months, and 80.1% of patients (29/36) continued immunotherapy. Among them, 12 cases were PSPD, accounting for 52% of the early imaging PD. The median time to PSPD was 5.7 weeks. Interestingly, the incidence of PSPD was 14.8% (9/61) in the PD-1 antibody–alone group while it was 4.8% (3/62) in the PD-1 antibody plus anticytotoxic T-lymphocyte-associated protein 4 (anti-CTLA-4) antibody group. A systematic review had also reported that Immune-based Response Evaluation Criteria in Solid Tumors standards have no significant impact on ORR and DCR statistics compared with RECIST 1.1, and the prediction difference of the mean survival time is also negligible (0.46 months) (82). Therefore, the current evaluation criteria and methods of neoadjuvant anti-PD-1/PD-L1 therapy efficacy need to be improved, which should also be a key consideration in the design of relevant clinical studies.

## Safety of neoadjuvant programmed cell death 1/programmed cell death ligand 1 inhibitors in colorectal cancer

With the wide application of immunotherapy in the field of cancer, more and more studies were reported concentrating on the safety in clinical practice. The immune-related adverse events (irAEs) might involve multiple organs including skin (like vitiligo), endocrine system (like hyperthyroidism), respiratory system (like pneumonia), gastrointestinal system (like diarrhea and colitis), and cardiovascular system (like myocarditis) (83, 84). The mentioned adverse events above usually occur in the first 2–3 months, while skin manifestations happen firstly (85). Even though the occurrence of irAEs might be associated with a clinical benefit for patients receiving anti-PD-1/PD-L1 therapy, grade 3–4 irAEs might be life-threatening and result in the permanent suspension of medication (86, 87). According to reported clinical trials, the incidence of grade ≥3 irAEs was 13%–22% using ICI monotherapy, while it was 22%–64% by dual ICIs (88). The overall adverse event rate reported in KEYNOTE 177 was 22% (33/153) with 9% (14/153) of grade ≥3 in the pembrolizumab group, while it was 13% (18/143) and 2% (3/143) in the chemotherapy group (65). The reported adverse events include hypothyroidism, colitis, hyperthyroidism, pneumonitis, adrenal insufficiency, hepatitis, infusion reactions, severe skin reactions, and thyroiditis after treating with pembrolizumab. Additionally, there are also literatures that support the fact that single PD-1/PD-L1 inhibitors caused fewer treatment-related adverse events than chemotherapy alone (22). At present, many academic organizations including the European Society for Medical Oncology, American Society of Clinical Oncology, and NCCN have published standards and guidelines for irAEs, which can escort the clinical use (89–91). Overall, these irAEs caused by PD-1/PD-L1 inhibitors are acceptable, predictable, and controllable. Therefore, anti-PD-1/PD-L1 therapy may play a role in all scenarios where neoadjuvant therapy can be used in the treatment of CRC, while safety is a premise and guarantee.

## Controversy and challenges

There are many environmental, dietary, and lifestyle factors including diet, smoking, alcohol, obesity, sleep, exercise, and microbiome that might influence the carcinogenic mechanisms, response to therapy, biology, and clinical outcome of CRC. These factors might influence the molecular pathology, immune infiltrates, and response to therapy in each patient differentially, which is increasingly evident in patients treated with immunotherapy. Additionally, gene-by-environment interactions also influence the germline genetic variations on both the immune system and cancer. Moreover, the molecular pathological epidemiology might be related to the microbiome, molecular pathologies, immune cell infiltrates, and clinical outcomes in CRC patients, especially in immunotherapy. Therefore, the relationship between the above-mentioned factors and neoadjuvant anti-PD-1/PD-L1 therapy for CRC still needs further exploration and discussion in the future.

Anti-PD-1/PD-L1 therapy is a useful therapeutic strategy following surgical resection, chemotherapy, radiotherapy, and targeted therapy, which perform great potential in the treatment of CRC. We should fully recognize the broad application prospect of immunotherapy in the neoadjuvant therapy of CRC in the future. However, the following points need to be noted. Firstly, the detection of the MMR/MSI status before CRC treatment is important, especially targeted detection combined with clinical characteristics, family history, and imaging features, to avoid missing the beneficiaries of immunotherapy. For pMMR/non-MSI-H patients, more novel neoadjuvant combination strategies like improving the immunogenicity and increasing the invasion ability of immune cells need to be monitored and developed. Secondly, as an emerging therapeutic method with potential, concerns about its safety still cannot be ignored. Especially, there are few studies on the evaluation of surgery-related complications after neoadjuvant anti-PD-1/PD-L1 therapy. Thirdly, the evaluation of treatment effect after neoadjuvant immunotherapy for CRC and the selection of following organ preservation and the Watch and Waiting strategy are not clear at present. Finally, it is urgent to explore the optimal mode of neoadjuvant therapy combined with immunotherapy for CRC, including the choice of radiotherapy mode (long course vs. short course), the cooperation of chemotherapy drugs, the choice of PD-1 medication timing (synchronous radiotherapy vs. sequential radiotherapy) whether total neoadjuvant therapy, and so on. In summary, it is reasonable that immunotherapy epical anti-PD-1/PD-L1 therapy may change the neoadjuvant therapeutic foreground of CRC and ultimately achieve the goal of patient benefit.

## Author contributions

All authors made substantial contributions to this review. ZZ, HY, and ZB conceived and designed the review. ZY, GW, XZ, JG, CM, YL, QW, LS, and PW retrieved and reviewed literatures. ZY and GW wrote the manuscript. ZZ, HY, and ZB reviewed and edited the manuscript. All authors read and approved the manuscript.

## Funding

This work was supported by grants from the National Key Technologies R&D Program (No. 2015BAI13B09), National Key Technologies R&D Program of China (No. 2017YFC0110904), and Clinical Center for Colorectal Cancer, Capital Medical University (No. 1192070313).

## Conflict of interest

The authors declare that the research was conducted in the absence of any commercial or financial relationships that could be construed as a potential conflict of interest.

## Publisher’s note

All claims expressed in this article are solely those of the authors and do not necessarily represent those of their affiliated organizations, or those of the publisher, the editors and the reviewers. Any product that may be evaluated in this article, or claim that may be made by its manufacturer, is not guaranteed or endorsed by the publisher.
